# Contribution of protein synthesis depression to poly-β-hydroxybutyrate accumulation in *Synechocystis* sp. PCC 6803 under nutrient-starved conditions

**DOI:** 10.1038/s41598-019-56520-w

**Published:** 2019-12-27

**Authors:** Kazuho Hirai, Miki Nojo, Yosuke Sato, Mikio Tsuzuki, Norihiro Sato

**Affiliations:** School of Life Sciences, Tokyo University of Pharmacy and Life Sciences, Hachioji, Tokyo 192-0392 Japan

**Keywords:** Polyhydroxyalkanoates, Environmental microbiology, Plant sciences

## Abstract

Poly-β-hydroxybutyrate (PHB) in cyanobacteria, which accumulates as energy and carbon sources through the action of photosynthesis, is expected to substitute for petroleum-based plastics. This study first demonstrated that PHB accumulation was induced, with the appearance of lipid droplets, in sulfur (S)-starved cells of a cyanobacterium, *Synechocystis* sp. PCC 6803, however, to a lower level than in nitrogen (N)- or phosphorus (P)-starved cells. Concomitantly found was repression of the accumulation of total cellular proteins in the S-starved cells to a similar level to that in N-starved cells, and a severer level than in P-starved cells. Intriguingly, PHB accumulation was induced in *Synechocystis* even under nutrient-replete conditions, upon repression of the accumulation of total cellular proteins through treatment of the wild type cells with a protein synthesis inhibitor, chloramphenicol, or through disruption of the *argD* gene for Arg synthesis. Meanwhile, the expression of the genes for PHB synthesis was hardly induced in S-starved cells, in contrast to their definite up-regulation in N- or P-starved cells. It therefore seemed that PHB accumulation in S-starved cells is achieved through severe repression of protein synthesis, but is smaller than in N- or P-starved cells, owing to little induction of the expression of PHB synthesis genes.

## Introduction

Photosynthetic microbial cells accumulate neutral lipids in organelles designated as lipid granules for storage of energy and carbon (C), and the accumulated level is elevated, depending on the progress of cell growth to the stationary phase or in response to environmental stimuli. A group of cyanobacteria, the postulated ancestors of plant plastids, have poly-β-hydroxyalkanoate (PHA) as neutral lipids in PHA granules, which is in contrast to eukaryotic microalgae that generally accumulate triacylglycerols (TGs) in lipid droplets^1,2^. The accumulated levels of PHA, however, depends on the type of stimulus in a cyanobacterial strain, and also on the cyanobacterial species when exposed to the same stimulus [reviewed in, e.g.,^3^]. Environmental stimuli for the induction of PHA accumulation in cyanobacteria include nitrogen (N)- or phosphorus (P)-starvation, high-intensity light, and supplementation of organic-carbon sources. In *Spirulina maxima*, e.g., addition of acetate to the culture, which enables mixotrophic cell growth, led to greater accumulation of poly-β-hydroxybutyrate (PHB) as PHA to 3% of dry cell weight (DCW), as compared with nutritional stresses such as N- or P-starvation that only slightly elevated it to 1%^4^. Meanwhile, *Aulosira fertilissima*, as compared with *S. maxima*, showed a much more remarkable increase of PHB to ca. 10% of DCW under N- or P-starved conditions^5^.

PHA, a bioplastic, is comparable to polypropylene, i.e., a petroleum-based plastic, in chemical and physical properties, and is also biodegradable. PHA with these features therefore is expected to compensate for future exhaustion of petroleum, and current plastic pollution, like the microplastic form in the ocean, through its substitution for petrochemical-based plastics^6^. In this context, development of a renewable system for PHA production using cyanobacteria that can grow vigorously in a photoautotrophic manner, i.e., with no input of an organic carbon-source, is attractive from both carbon-neutral and economic aspects^7^. So far, a thermophilic cyanobacterium, *Synechococcus* sp. MA19, has shown the highest PHA accumulation and production yield values, which reached 62 weight% in DCW and 9.2 mg•L^−1^•h^−1^, respectively, during exposure of photoautotrophic cells to phosphate-limited conditions^8^. However, the PHA production yield, which is the core factor for its commercialization, is 60-fold lower in *Synechococcus* sp. MA19 than in heterotrophic hyper-PHA producing bacteria like *Aeromonas hydrophila* 4AK4 (540 mg PHA•L^−1^•h^−1^), which has allowed no choice other than to utilize heterotrophic *Cupriavidus necator* for PHA production even at the expense of organic-source input^9,10^. It thus seems that, for economization of commercial PHA production, cyanobacteria have to be genetically activated as to PHB synthesis, however, information is limited on the metabolic processes that play key roles in PHA accumulation, which could become the targets of genetic manipulation.

The bacterial genes for PHA synthesis includes *phaA* for ß-ketothiolase that catalyzes the condensation of two molecules of acetyl-CoA for the synthesis of acetoacetyl-CoA, *phaB* for NADPH-dependent acetoacetyl-CoA reductase that synthesizes the monomeric precursor, hydroxyalkanoate, and the gene(s) for PHA synthase that polymerizes the precursor^11^. PHB synthases can be divided into four classes according to the primary sequences of the subunit proteins: Classe I and II PHA synthases are encoded by the genes for single subunits, *phaC*, and *phaC1* or *phaC2*, respectively, while class III and IV PHA synthases are encoded by the genes for heterodimer subunits, *phaC* and *phaE*, and *phaC* and *phaR*, respectively^12^. It is generally accepted that class I, III, and IV PHA synthases predominantly utilize short-chain length monomers (C3-C5), in contrast, class II PHA synthase having a preference for medium-chain length monomers (C6-C14,^12^).

*Synechocystis*, despite its relatively low ability as to conditional PHB synthesis, has been intensively investigated regarding the mechanism of PHB synthesis, particularly under N-starved conditions^13,14^, owing to established molecular-biological tools such as genomic DNA databases and genetic manipulation^15^. The genes responsible for PHB synthesis in *Synechocystis*, so far identified, includes *phaA*, *phaB*, and *phaC* and *phaE* for class III PHA synthase that polymerizes the precursor, D-3-hydroxybutyryl-CoA^16,17^. Through these studies, it was found that the mechanism of N-starvation induced PHB accumulation includes up-regulation of the levels of transcripts and/or translated proteins as to the genes for the synthesis of polyhydroxybutyrate (PHB), the sole PHA in *Synechocystis*, consistent with a concomitant elevation in PHB synthesis activity^18,19^. In addition, the Sll0783 protein contributes to the activation of PHB synthase at the posttranscriptional level^18^. Meanwhile, from the aspect of carbon metabolism, it has been recently shown that glycogen catabolism and following glycolytic pathways are important for PHB accumulation in *Synechocystis* under N-starved conditions^20–22^.

We previously observed that TG accumulation was induced in the wild type cells of a green alga, *C. reinhardtii*, on treatment with an inhibitor of protein synthesis, or in disruptant cells as to the *ARG9* gene for Arg synthesis, both observations therefore implying the responsibility of repression of global protein synthesis for TG accumulation^23^. In view of that total cellular proteins occupy ca. 50% of dry cell matter in aquatic photosynthetic organisms^24^, it was reasoned that TG accumulation is facilitated through diversion of metabolic carbon-flux from the synthesis of proteins to that of other C-metabolites including TG. Accordingly, this notion, together with the observed up-regulation of the genes involved in TG synthesis at the transcript level, could explain TG accumulation in N- or S-starved *C. reinhardtii* cells, which become defective in the synthesis of general amino acids and that of S-containing amino acids, Cys and Met, respectively, for protein synthesis^23^. A similar metabolic mechanism might function for PHB accumulation in *Synechocystis* under N-starved conditions, however, the quantitative behavior of total cellular proteins thus far has hardly correlated well with PHB accumulation in N-starved cells. Moreover, contrary to the above notion, S-starvation, distinct from N-starvation, showed no induction of PHB accumulation in *Synechocystis* sp. PCC 6803^25^. There is thus no convincing explanation of how the level of PHB accumulation is determined depending on the type of nutrient stress in cyanobacteria. This issue should be addressed through characterization of PHB accumulation from metabolic and genetic aspects in a cyanobacterial strain grown under identical conditions except for depleted nutrients.

Here, the accumulated levels of PHB were compared between *Synechocystis* cells growing under well-defined conditions for starvation of S, N, and P, respectively. Also compared were changing patterns of the content of total cellular proteins or the levels of the respective transcripts of PHA synthesis genes. Furthermore, the role of repressed protein synthesis in the induction of PHB accumulation was directly investigated in *Synechocystis* through genetic and metabolic manipulation of global protein synthesis. The distinct accumulation patterns of PHB in cells limited in the above three macronutrients will be discussed with regard to the repression levels of accumulation of total cellular proteins and also to the induced expression levels of the PHB synthesis genes.

## Materials and Methods

### Cyanobacterial material and culture conditions

The cyanobacterial strains used were *Synechocystis* and its disruptant as to the *argD* gene (see below). The wild-type cells were cultured at 30 °C in a glass tube containing BG11 medium, with illumination (10 W·m^−2^) and aeration^26^. The wild-type cells were pre-cultured until the OD730 value became ca. 0.3 to 0.4 with a spectrophotometer (DU 640, Beckman), and thereafter they were shifted to N-, P- or S-starved conditions for further growth with the OD730 value adjusted to 0.3. The *argD*-disruptant cells were cultured under nutrient-replete conditions with supplementation of 1 mM citrulline for normal growth, and thereafter they were shifted to citrulline-free medium for further growth with Arg synthesis repressed.

### Disruption of the *argD* gene or *phaAB* operon in *Synechocystis*

For amplification of a DNA fragment including the coding region of the *argD* gene in *Synechococcus*, PCR was performed with EX-taq DNA polymerase, with the use of primer set 1 (Supplementary Table S1), as we previously described^26^. A product of 1.5 kbp was ligated to the pMD19 vector (Takara), and the resultant plasmid was used as a template to amplify a DNA fragment with Kod-plus DNA polymerase, with the use of primer set 2. The DNA product contained pMD19 with the two DNA fragments, which corresponded to the N- (0.54 kbp) and C-terminal (0.51 kbp) regions, linked to the respective ends. This DNA product was ligated with the chloramphenicol-resistant gene cassette (1.1 kbp) obtained from pCMT9 by SmaI digestion. The resultant plasmid containing the disrupted *argD* gene was used to transform wild-type (WT) cells of *Synechocystis* by homologous recombination, as we described previously^26^.

### Determination of OD730 values, the respective contents of Chl and phycobilisome, and total cellular proteins

Aliquots of the culture of the wild-type or *argD*-disruptant were taken at the indicated times for measurement of the contents of Chl *a* and phycobilisome, as we previously described^26,27^.

### Extraction and quantification of PHB

Dried cells were subjected to extraction of pigments including Chl in a mixed solvent of chloroform and methanol (2:1, by vol.), and then to PHB extraction in chloroform at 60 °C for 48 h, according to the method described in^28^. The solution of PHB in chloroform was filtrated through a glass fibre membrane, and thereafter 2 vol. of cooled diethyl ether was added to the filtrate. The resultant solution was cooled in a refrigerator for precipitation of PHB, which was then collected through filtration on a glass fibre membrane. The membrane in which PHB was collected was placed in a test tube for air-drying, and thereafter heated in H_2_SO_4_ at 100 °C for 10 min. The quantity of crotonic acid generated was determined through spectroscopic measurement at 235 nm. PHB purchased from Sigma was used for preparation of standards for the quantitative measurement.

### Microscopic observation of intracellular PHB granules

The culture of cyanobacterial cells (100 μl) was centrifuged to prepare the pellet of cells, which were then resuspended in 80% methanol. The cell suspension was left to stand for 1 min at room temperature, and centrifuged to obtain cell pellet where Chl was extracted. This Chl extraction step was repeated until the color of Chl disappeared in the supernatant. The cells where Chl was extracted were resuspended in distilled H_2_O (50 μl) and stained with a Nile red solution (0.5 mg/mL in acetone; 1 μl) for 1 min. The cell suspension was centrifuged to pellet the stained cells, which was then washed with distilled H_2_O for the removal of the Nile red solution. The obtained cell pellet was resuspended in distilled H_2_O (5–20 μl), and used for fluorescence microscopic observations (BX-53; Olympus Optical Co., Tokyo, Japan) with the use of an excitation filter BP530-550 (530–550 nm) and an emission filter BA575IF (>575 nm).

### Quantitative PCR analyses of transcripts

Quantitative real-time (RT) PCR was performed with RNA prepared from WT *Synechocystis* cells, with the use of a Rotor-Gene Q PCR System (Qiagen), as described in^29^. Primer sets 3–6 in Supplementary Table S1 were used for amplification of the sequences related with PHB synthesis genes, *phaA-C*, and *phaE*, respectively. Meanwhile, RNA was also subjected to RT-PCR with primer sets 7–9 for *glnB*, *sbpA*, and *phoA* that are induced under N-, S-, and P-starved conditions, respectively (Supplementary Table S1). The expression levels of the respective genes were normalized as to that of *rnpB*, the gene for a subunit of ribonuclease P, with the use of primer set 10.

## Results

### Effects of nutrient-starvation on cell growth, protein contents, and PHB in *Synechocystis*

**Figure 1:**
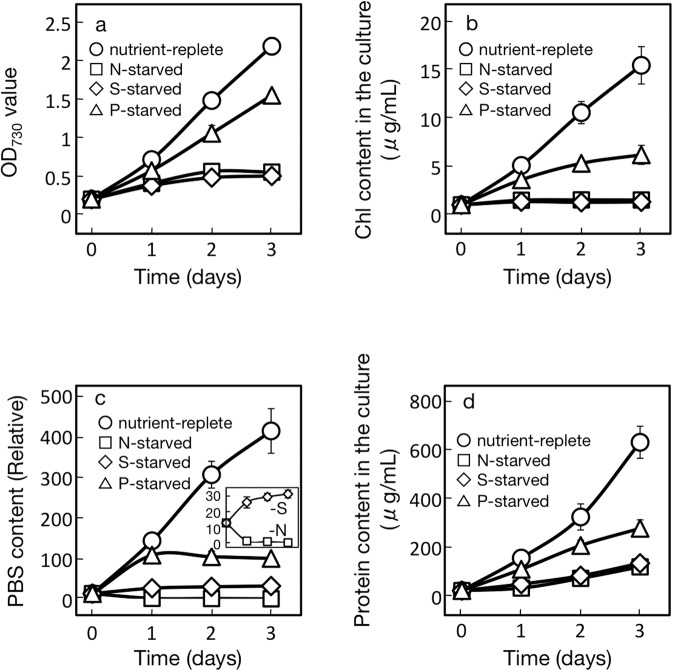
Effects of S-, N- or P-starvation on cell growth, and the Chl, PBS and total cellular proteins contents in Synechocystis. Optical density (OD_730_, **a**) was monitored as reflecting cell growth in the culture, whereas Chl (**b**), PBS (**c**), and total cellular proteins (**d**) were measured quantitatively. The inset in (**c**) shows the changes of PBS contents in S- and N-starved cells on a magnified scale to clearly exhibit the distinct patterns in these two types of cells. Cells were grown, respectively, under nutrient-replete (circles), S-starved (diamonds), N-starved (squares), and P-starved (triangles) conditions. The values are the averages ± SE for three distinct biological replicates.

**Figure 2:**
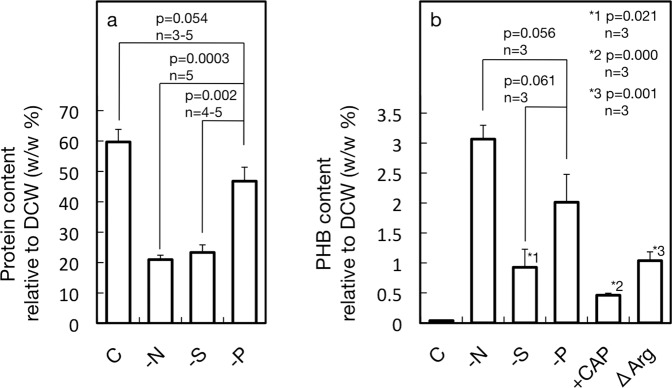
Effects of S-, N- or P-starvation on the protein and PHB contents. Total cellular proteins (**a**) and PHB (**b**), relative to DCW, were quantitated. The values are the averages ± SE for three distinct biological replicates. The significance of differences was evaluated by means of Student’s *t*-test. *1–3 respectively indicate the significance of differences from the control level under S-replete conditions.

*Synechocystis* cells were cultured under identical conditions except for nutrient starvation to examine the dependency of the PHB accumulation level on the type of nutrient stress. Here, the possibility was examined that PHB accumulation is brought about through repression of the net synthesis of total cellular proteins under nutrient-starved conditions, in view of the functionality of such a metabolic mechanism for algal TG accumulation^23^. We first compared physiological aspects, including the accumulation of total cellular proteins, between S-, N-, and P-starved cells. As a result, it was found that S-, N- or P-starvation, respectively, caused *Synechocystis* cells to show delayed growth, with S- and N-starvation exerting more deleterious effects than P-starvation (Fig. 1a). A similar nutrient-dependent delay was also observed with a Chl- or phycobilisome-accumulation pattern (Fig. 1b,c), reminiscent of a previous report of the use of another cyanobacterium, *Synechococcus* sp. PCC 7942^30^. Furthermore, a well-known bleaching process through PBS degradation in N-starved cells, but in neither S- nor P-starved ones, was confirmed (Fig. 1c inset,^31^). Above all, it was demonstrated that S-, N-, or P-starved *Synechocystis* cells were impaired in protein accumulation, with S- or N- starvation having more serious effects than P-starvation, which was consistent with the above nutrition-dependent growth injury patterns (Fig. 1d). Accordingly, the protein content per dry cell weight (DCW), which amounted to 60% in control cells, was markedly reduced to 21 and 23% in S- and N-starved cells, respectively, and less severely to 47% in P-starved ones (Fig. 2a).

**Figure 3:**
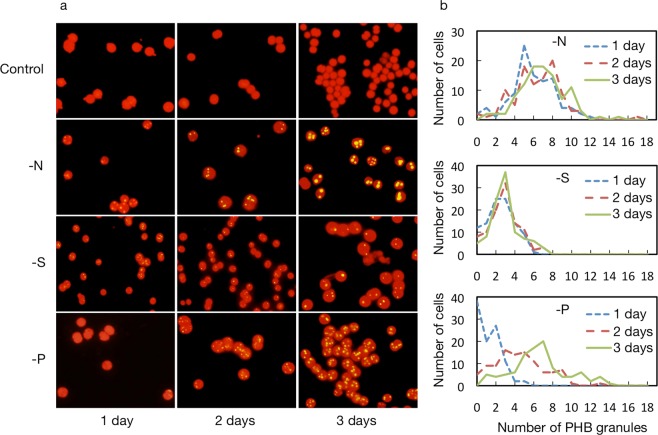
PHB granules accumulation patterns depending on depleted nutrients. PHB granules, which were stained with Nile-red, appeared as yellow granules in the respective nutrient-starved cells. The background shows the red autofluorescence of cellular lipids after the extraction of Chl.

Microscopic analysis clearly revealed distinct accumulation patterns of PHB granules in cells starved of S, N, and P (Fig. 3a,b): in a day after the onset of S-starvation, PHB granules, which emitted yellow fluorescence, abruptly appeared, in the background of red fluorescence from lipids of Chl-extracted cells^32^. The number of PHB granules was 2.5 on average, and stayed almost the same (3.0) for the next 2 days. PHB granules also rapidly appeared in a day in N-starved cells, but to a much higher level of 5.9 in number on average, which remained almost unaltered for the next two days (6.4–6.9). In contrast, P-starved cells were slow to show PHB granules such that as many as ca. 40% of the cell population had no visible PHB granules in a day, which resulted in a much smaller number of PHB granules of 1.3 on average. The number in P-starved cells, however, steadily increased to 6.6 on average in three days. In accordance with the PHB granules numbers, PHB finally amounted for 0.9% of dry cell weight in S-starved cells on day 3 whereas it reached higher levels in the other starved cells, i.e., 3.1 and 2.1% in N- and P-starved cells, respectively (Fig. 2b). It thus seems likely that the PHB granules numbers reflect the intracellular PHB content, which allows us to compare the trends of the quantitative changes in PHB in the three types of nutrient-starved cells. Thus far, it has been demonstrated that N- and P-starvation induce PHB accumulation to almost similar levels in photoautotrophically growing cells of cyanobacteria including *Synechocystis*, however, there has been no detailed argument about the accumulation process^5,14^. This study found the markedly delayed induction of PHB accumulation in P-starved cells, as compared with in N-starved ones, which was enabled through relatively short-term observation. Above all, our results definitely confirmed that S-starvation, as well as N- and P-starvation, is a stressor for induction of PHB accumulation in *Synechocystis*. Collectively, it was found that PHB accumulation was accompanied by repression of protein synthesis to reduce the contents of total cellular proteins under the nutrient-starved conditions examined here, implying some relationship between the two metabolic processes. Importantly, it is of note that PHB granules was generated to some extent even at the early phase under S- and N-starved conditions, concomitantly with severe repression of cellular protein accumulation, but not under P-starved ones where protein accumulation was less deleteriously affected.

### Induction of PHB granules formation and PHB accumulation through repression of protein synthesis

**Figure 4:**
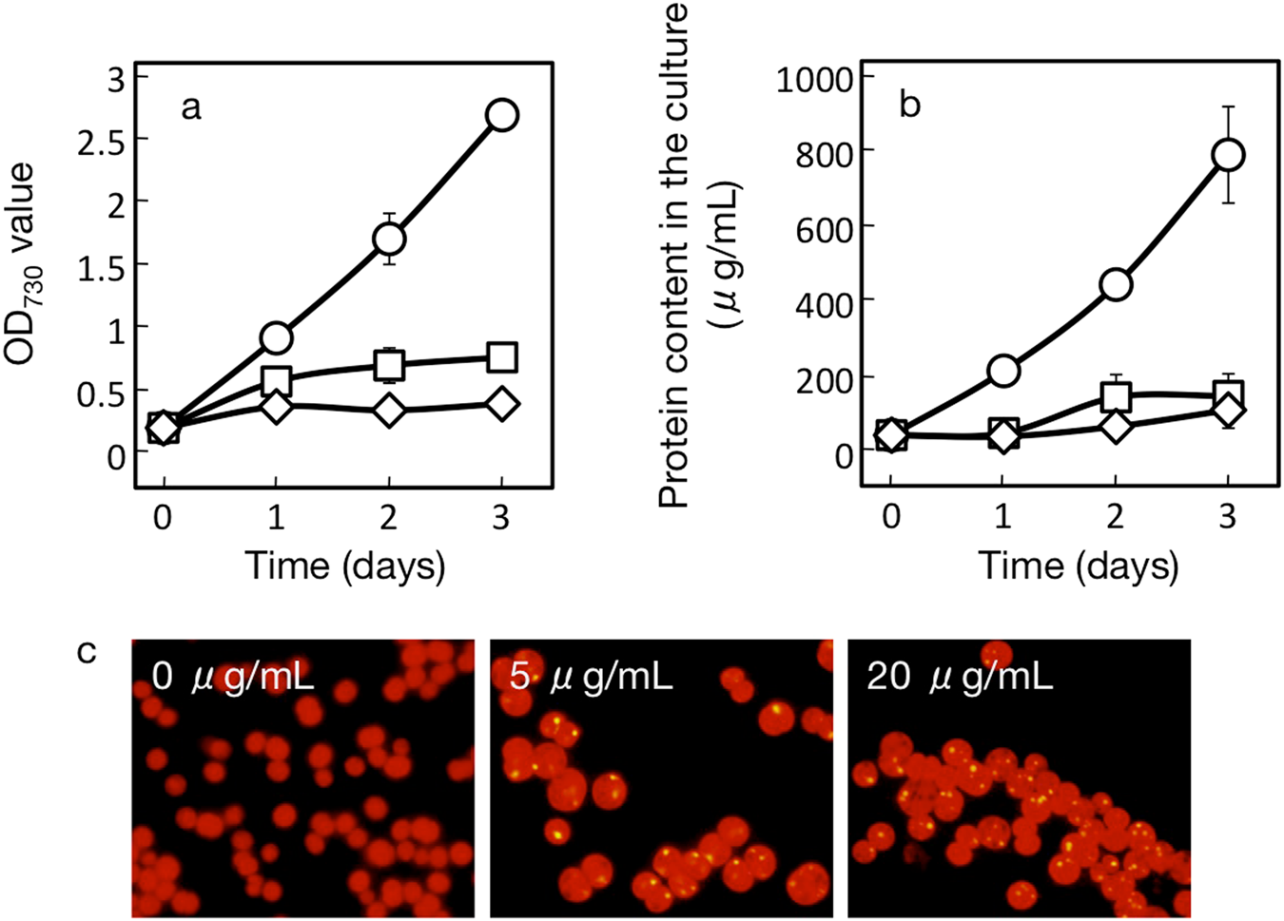
Effects of CAP, an inhibitor of protein synthesis, on cell growth, protein contents and PHB granules accumulation. Cells were grown under nutrition-replete conditions in the presence of CAP (5 and 20 mg/ml culture) or its absence, for measurement of OD730 (**a**) and total protein contents (**b**), and also for fluorescent-microscopic observation of PHB granules (**c**). Circles, squares, and diamonds indicate the addition of 0, 5 and 20 mg CAP/mL culture, respectively, in (**a,b**). The values are the averages ± SE for three distinct biological replicates.

To evaluate the contribution of the repressed protein synthesis to simulation of PHB accumulation in nutrient-starved cells, we attempted to specifically decrease the cellular protein level in *Synechocystis* under nutrient-replete conditions with the use of a metabolic inhibitor or through genetic manipulation. WT cells of *Synechocystis*, upon treatment with chloramphenicol (5 or 20 µg·mL^−1^), an inhibitor of translation by 70S ribosomes, were retarded as to both growth and total protein accumulation (Fig. 4a–c). In line with this, PHB was found to accumulate to 0.5% DCW even under nutrient-replete conditions, concomitantly with the appearance of PHB granules (Fig. 2b).

**Figure 5:**
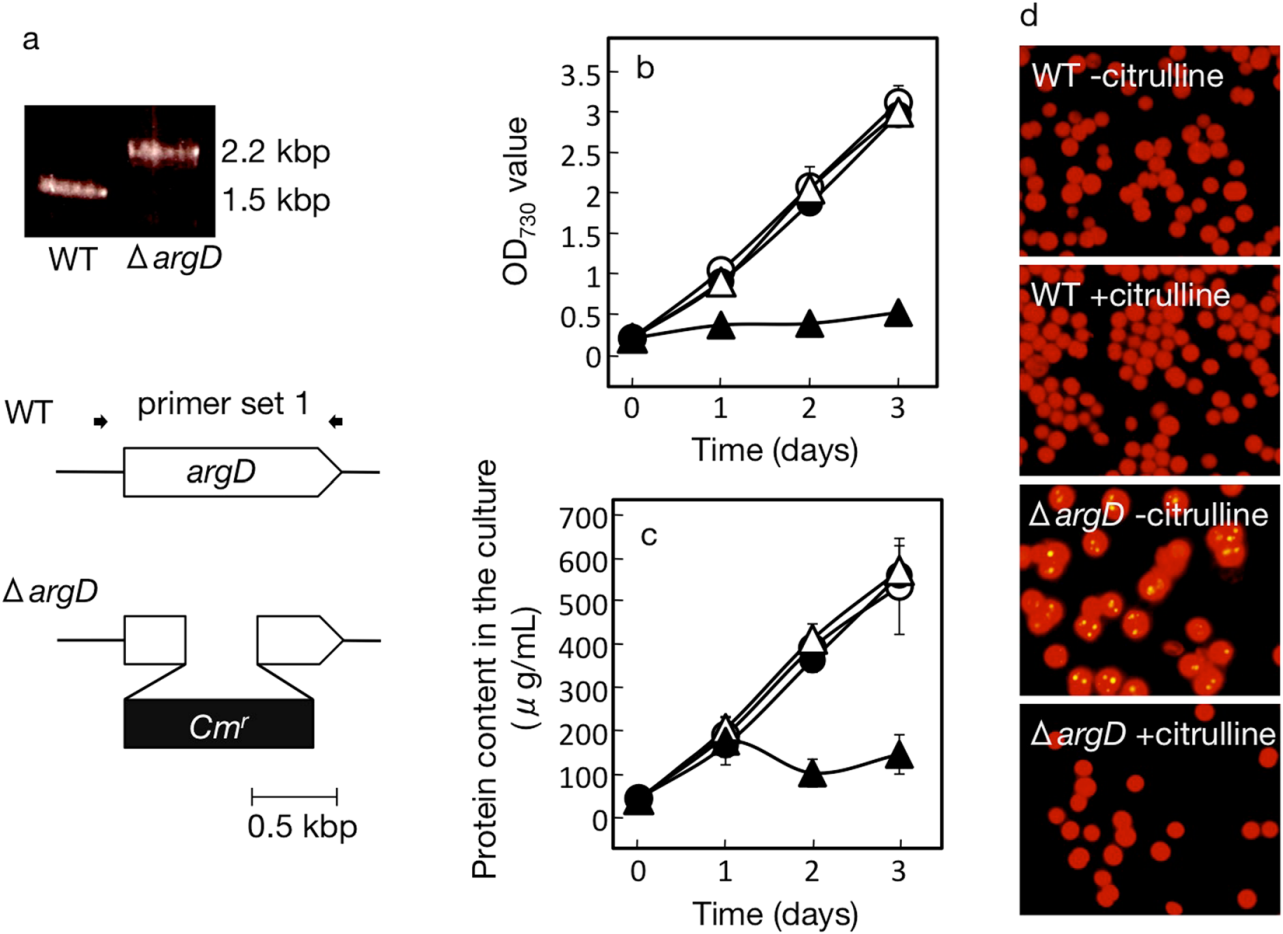
Disruption of *argD* for repression of protein synthesis and its effects on PHB accumulation. (**a**) The strategy for *argD* disruption and its confirmation by PCR with the use of primer set 1 (Supplementary Table 1). WT and *ΔargD* disruptant cells, which had been grown in the presence of citrulline, were transferred to new culture medium with or without the supplementation of citrulline, for measurement of OD_730_ (**b**) and total protein contents (**c**). Circles and triangles indicate WT and *ΔargD* cells, respectively. Closed and open symbols indicate cells grown with citrulline and without it, respectively. (**d**) Fluorescent-microscopic observation of PHB granules in WT or *ΔargD* cells grown for three days in the presence or absence of citrulline.

In parallel, we generated a mutant of *Synechocystis* that is defective pin-pointedly in Arg synthesis, and, inevitably, in global protein synthesis, through disruption of the *argD* gene for N-acetylornithine aminotransferase (∆*argD*, Fig. 5). As was shown on PCR-based analysis, the DNA fragment amplified with PCR primer set 2 (Supplementary Table S1) for the *argD* gene was 0.7-kbp longer in the mutant than in the wild type, owing to the insertion of a 1.1-kbp chloramphenicol-resistant cassette, concomitantly with excision of a 0.4-kbp fragment from *argD* (Fig. 5a). The ∆*argD* disruptant, compatible with a previous report^33^, was lethal due to its inability to synthesize Arg, however, it grew as vigorously as the WT, with supplementation of citrulline, which enables the synthesis of Arg through recovery in the functionality of the urea cycle (Fig. 5b). The disruptant was then pre-cultured with supplementation of citrulline under normal nutrient-replete conditions, and thereafter subjected to removal of the citrulline from the culture medium. Upon this removal, cell growth was markedly delayed with little increase in the content of total cellular proteins, which was accompanied by PHB granules appearance and PHB accumulation to 1.0% DCW (Figs. 2b and 5b–d). In contrast, citrulline-removal in the wild-type culture little impacted cell growth or total cellular protein synthesis, accordingly, PHB granules accumulation was never induced (Fig. 5b–d). Collectively, we confirmed that repressed protein synthesis directly or indirectly induces PHB accumulation in *Synechocystis*, through both metabolic and genetic manipulation. Probably, the functionality of a pre-existing PHB synthesis system, including the responsible enzymes encoded by *phaA*, *B*, *C*, and *E*, would be high enough to rewire C-flux for PHB accumulation to the observed levels under these manipulated conditions. It was therefore supposed that the decreased protein synthesis is one of the important factors for PHB accumulation in the nutrient-starved cells examined in this study.

### Nutrient-dependent induction of PHB synthesis gene expression

**Figure 6:**
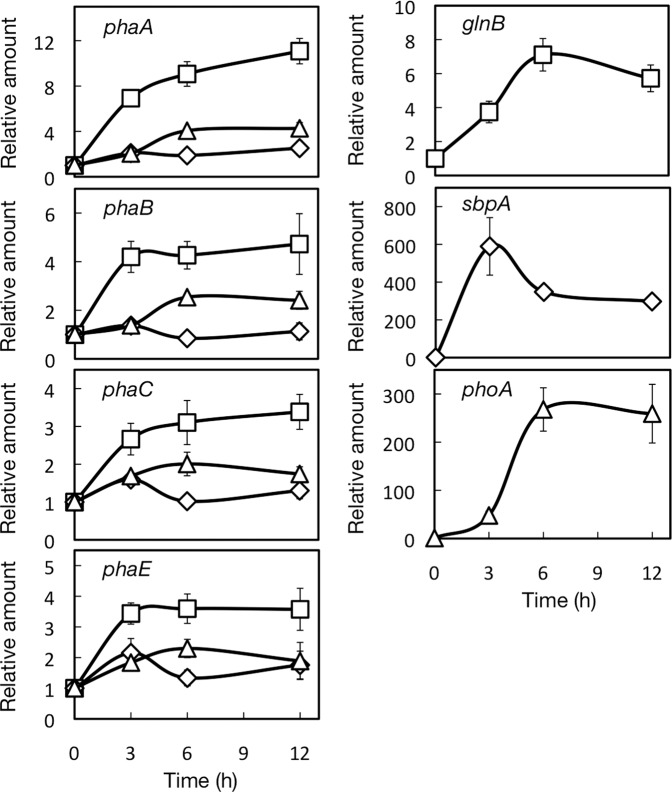
Dependency of starvation induced expression of PHB synthesis genes on nutrient species. Changes in the transcript levels of *phaA*, *B*, *C* and *E* were monitored through RT-PCR in *Synechocystis* cells after their transfer to S-, N- and P-starved conditions. As positive controls, known expression induction as to *sqbA*, *glnB*, and *phoA* was confirmed under S-, N- and P-conditions. Diamonds, squares and triangles indicate S-, N- and P-starved cells, respectively.

S-starvation, as compared with N-starvation, less strongly induced PHB accumulation despite their similar effects on the repression of protein accumulation, which indicated some other factors underlie determination of the accumulated level of PHB (Fig. 2a,b). Previous reports that demonstrated the induction of the expression of PHB synthesis genes in N-starved cells prompted us to compare the expression levels of these genes between cells starved for the respective nutrients^18,19^ (Fig. 6). Here, N-starved cells showed that the mRNA level of the *phaA* or *phaB* gene steeply increased to reach more than 10- and 4-fold of the initial levels, respectively, in 12 h, while that of *phaC* or *phaE* showed a more than 3-fold increase in the same time. In contrast, S-starvation showed only small effects, if any, as to induction of the expression levels of the PHB synthesis genes. Meanwhile, P-starved cells, like N-starved ones, definitely showed elevated mRNA levels of *phaA* and *phaB*, although to a lesser extent. It is likely that up-regulation of PHA synthesis genes as to their transcript levels contributed to the PHB accumulation in N- and P-starved cells, but not in S-starved ones.

## Discussion

PHAs are typical neutral lipids in a group of cyanobacteria, however, information is limited on metabolic processes that are important for PHA accumulation. This study compared the accumulation mechanisms for PHB in *Synechocystis* between cells starved of S-, N-, and P-sources. First of all, we demonstrated that S-starvation is one of the stressors for induction of the accumulation of PHB in *Synechocystis*, together with conventional observation of PHB accumulation in N- and P-starved cells of it^13,14^. Thus far, increased levels of PHB accumulation upon N-starvation have been correlated to the induced expression of PHB synthesis genes. Apart from this ordinary aspect, it was successfully verified here that repression of protein synthesis directly or indirectly contributes to the enhancement of the level of PHB accumulation through both genetic and metabolic manipulation under nutrient-replete conditions, i.e., disruption of *argD*, and application of CAP to the WT cells. It seems reasonable that the diversion of metabolic carbon flux from the synthesis of proteins to that of the other C compounds including PHB is another crucial factor for PHB accumulation. We therefore propose that the level of PHB accumulation should be interpreted on the basis of both the induced expression levels of PHB synthesis genes and the repressed level of total cellular protein accumulation.

S-starved cells, as compared with N-starved ones, exhibited lower accumulation of PHB granules and PHB throughout the starvation period, despite similar repression levels for protein accumulation in both cells (Figs. 2b and 3). These results can be accounted for by no or much weaker induction of the expression of PHB synthesis genes under S-starved conditions than under N-starved ones (Fig. 6). Strong induction of PHB synthesis genes upon N-starvation would lead to efficient utilization of diverted C-flux for PHB accumulation (3.1% DCW, Figs. 2b and 3), which was reminiscent of the mechanism of TG accumulation based on the expression induction of TG synthesis genes in S- or N-starved *C. reinhardtii* cells, i.e., under conditions where protein synthesis was repressed^23^. Meanwhile, the low PHB content in S-starved cells (0.9% of DCW) was close to that with Δ*argD* (1.0% DCW) or that in WT cells with administration of CAP (0.5% DCW), suggesting that PHB accumulation in S-starved cells might be explained largely by both repression of protein synthesis and the functionality of preexisting PHB synthesis enzymes.

On the other hand, P-starved cells, as compared with S- or N-starved ones, exhibited lower PHB granules accumulation at the early phase of starvation, which might be due to both weak repression of protein accumulation and no strong expression of PHB synthesis genes (Figs. 1d, 3 and 6). The P-starved cells, however, showed an enhanced PHB granules accumulation level after prolonged exposure to P-starvation, which resulted finally in PHB accumulation to 2.0% DCW (Fig. 2b). This delayed, but relatively higher accumulation of PHB granules or PHB in P-starved cells than in S-starved ones, would be caused probably by the stronger induction of PHB synthesis genes in P-starved cells, which might compensate for the weaker repression of protein synthesis. The PHB accumulation level in P-starved cells, which fell short of that in N-starved cells, would be brought about by both weaker repression of protein synthesis and lower induced expression levels of PHB synthesis genes in P-starved cells than in N-starved ones.

It was previously implied that an increase in the intracellular ratio of [NADPH]/[NADP^+^] supports PHB synthesis under N-starved conditions in *Synechocystis*^34^ and a heterotrophic bacterium, *Alcaligenes eutrophus*^35^. This over-reduced state is generated through impairment of metabolism in general^36^, as reflected by severe growth defects in N-starved *Synechocystis* cells (Fig. 1). The metabolism that would be impaired includes the consumption of reducing power, e.g., reduction of nitrate to ammonia for nitrogen assimilation, which intrinsically utilizes 30% of the reducing power produced through photosynthetic electron transport^37^. It should be emphasized, however, that the PHB synthesis system would be able to consume the resultant surplus reducing power only when C-flux is rewired into this system. Meanwhile, it was previously proposed that, in P-starved cyanobacterial cells, PHB accumulation is promoted by an increasing ratio of [NADPH]/[ATP]^38^, which should also be accompanied by rewired C flux. The metabolic mechanism of PHB accumulation from the aspect of C-flux, as discussed above, is thus compatible with ones previously proposed on the basis of the cellular energetic state.

Meanwhile, the possibility is raised that induced accumulation of storage C compounds through consumption of surplus NADPH represses the generation of toxic reactive oxygen species (ROS) for cell survival, as was shown for TG synthesis in N-starved *C. reinhardtii* cells^23^. However, it was not the accumulation of PHB, but that of glycogen that is responsible for cell survival in *Synechocystis* under N-starved conditions, which was demonstrated through characterization of the respective mutants deficient in PHB- and glycogen-synthesis^39^. The authors concluded that glycogen, which occupied much more DCW (25%), in comparison to PHB (8%), plays a predominant role as an energy sink^38^. It might be that, under experimental culturing conditions, PHB synthesis is generally dispensable in cyanobacteria such as *Synechocystis* and *Spirulina platensis*, which show only low PHB accumulation (less than 10% DCW). It remains for a future study to investigate the physiological role of PHB in high-PHB producing cyanobacteria like *Synechococcus* MA19^8^.

Overall, it could be concluded that a higher accumulation level of PHB is achieved by rewiring of greater C-flux into the PHB synthesis pathway, through both repression of protein synthesis and activation of the PHB synthesis system. This conclusion was convincingly confirmed through our experimental system with identical growth conditions except for the depleted nutrients only. With the use of a well-defined growth system similar to that used in this study, transcriptomic analysis of a cyanobacterium, *Synechococcus* sp. PCC 7002, previously revealed general and specific responses to S-, N-, and P-starvation, concerning regulatory gene expression at the transcript level^40^. Intriguingly, the expression levels of the genes for the protein subunits of ribosomes, i.e., the translational machinery, were markedly down-regulated under the respective three starvation conditions, whereas that for the small subunit of ribulose 1,5-bisphosphate carboxylase/oxygenase responsible for CO_2_ fixation was severely down-regulated only under N- and S-starved conditions with no effect under P-starved ones^40^. These results seemed compatible with our ones where the accumulation of total cellular proteins was repressed more severely in N- and S-starved cells than in P-starved ones (Fig. 2a). The regulatory mechanism of PHB accumulation dependent on the type of nutrient starvation will be more comprehensively understood through future characterization of the expression patterns of the genes for individual proteins in *Synechocystis* cells starved of the respective nutrients.

PHA produced by photoautotrophic cells of cyanobacteria has been expected to be a material for biodegradable and renewable bioplastics that will substitute for petro-chemical plastics for sustainability of global environments. One of the successful ways to improve PHB production in *Synechocystis* was to disrupt the genes responsible for the synthesis of a C- and chemical-energy storage compound, glycogen, thereby rewiring metabolic C-flux into PHB synthesis^39,41^. On the basis of our observations, it is expected that repression of protein synthesis to an extent that is less great than for N-starvation, but allows substantial C-flux deviation, could be compatible with continuous growth of cells with highly improved PHB production. Such devices might be constructed in *Synechocystis* cells through introduction of leaky mutations as to the genes for amino acid synthesis like *argD* or those for the subunit proteins of the translation machinery. Further enhancement of PHB synthesis ability in *Synechocystis* will be achieved through the combination of such leaky mutations with other genetic modifications that have been verified to be effective, including disruption of glycogen synthesis genes, and overexpression of PHB synthetic and/or glycolytic genes^20,39,41,42^. Such genetic modification of *Synechocystis* for elevation of PHB accumulation to its potentially ultimate level would be a model for genetic improvement of the ability of high-PHA producing cyanobacteria to a commercial level.

In conclusion, this study showed that sulfur-starvation is a novel stressor for induction of PHB accumulation in *Synechocystis*. It was also found that repression of accumulation as to total proteins in the cells, which are the major cellular C-compounds, can induce PHB accumulation through deviation of C-metabolic flow from accumulation of total proteins. Accordingly, it could be proposed that PHB accumulation patterns, which are specifically characteristic for the cells starved for S, N, and P, respectively, should be interpreted based not only on an extent to which the PHB synthesis genes are induced, but also on that to which protein accumulation is repressed. Above results we obtained will be the foundation for a comprehensive understanding of the mechanism by which *Synechocystis* cells accumulate PHB. Such information will help greatly improve the ability of high-PHA producing cyanobacteria to a commercially expected level.

## Supplementary information

Supplementary Table S1
