# Supplementary material for: Contribution of protein synthesis depression to poly-β-hydroxybutyrate accumulation in Synechocystis sp. PCC 6803 under nutrient-starved conditions
Source: Sci Rep. 2019 Dec 27;9:19944. doi: 10.1038/s41598-019-56520-w (PMC6934822; doi:10.1038/s41598-019-56520-w)
Supplement: Supplementary file 1 — Supplementary Table S1 [file 41598_2019_56520_MOESM1_ESM.pdf]

Title: Contribution of protein synthesis depression to poly-β-hydroxybutyrate accumulation in *Synechocystis* sp. PCC 6803 under nutrient-starved conditions

Authors: Kazuho Hirai, Miki Nojo, Yosuke Sato, Mikio Tsuzuki, and Norihiro Sato

Supplementary Table S1 Primer sequences

	Forward	Reverse
primer set 1	CCCCTGGCGATCGATGAGATTTAA	TCAAACCAAAGTGGCGATCGCCTG
primer set 2	GGCAAGTTGTGGGGCTACGAACAT	AACCCGGTCCGCACAGGAATGCTC
phaA (*slr1993*)	GGAATCCATGTCCCAAACTG	AAACCATCATGGAGCAGGAG
phaB (*slr1994*)	GGCATGTATGAACGGAAAGC	CCAGCTTTAGTGGCGGAATA
phaC (*slr1830*)	TTTTGCTCAGGTGGGGTTAG	CCCACCTGGATGTCATCTTC
phaE (*slr1829*)	AATGCTGAACCTCCCTACCC	TTAGCCTGGGTTTGCTTCTG
glnB (*ssl0707*)	GCCGTCAAAAAGGTCAAACA	CCATGTCAACCTGTCCTTCG
sbpA (*slr1452*)	CATTCCCAAATTCACCGAAG	TCCGCTTCTAGACCATCCAC
phoA (*sll0654*)	GTTTCAGTCCTGTGCCCAAC	TGCACTTGACCAGCTTCTTG
rnpB	AAGAGCGCACCAGCAGTATC	ATTCCTCAAGCGGTTCCAC
